# Correlative Microscopy: a tool for understanding soil weathering in modern analogues of early terrestrial biospheres

**DOI:** 10.1038/s41598-021-92184-1

**Published:** 2021-06-17

**Authors:** R. L. Mitchell, P. Davies, P. Kenrick, T. Volkenandt, C. Pleydell-Pearce, R. Johnston

**Affiliations:** 1 Advanced Imaging of Materials (AIM) Facility, College of Engineering, Bay Campus, Swansea University, Swansea, SA1 8EN UK; 2 Earth Sciences Department, The Natural History Museum, Cromwell Road, London, SW7 5BD UK; 3 Sheffield Tomography Centre (STC), The University of Sheffield, North Campus, Broad Lane, Sheffield, S3 7HQ UK; 4 Carl Zeiss Microscopy GmbH, Carl-Zeiss-Straße 22, 73447 Oberkochen, Germany

**Keywords:** Element cycles, Biogeochemistry, Evolutionary ecology, Palaeoecology, Imaging, Microscopy

## Abstract

Correlative imaging provides a method of investigating complex systems by combining analytical (chemistry) and imaging (tomography) information across dimensions (2D-3D) and scales (centimetres-nanometres). We studied weathering processes in a modern cryptogamic ground cover from Iceland, containing early colonizing, and evolutionary ancient, communities of mosses, lichens, fungi, and bacteria. Targeted multi-scale X-ray Microscopy of a grain in-situ within a soil core revealed networks of surficial and internal features (tunnels) originating from organic-rich surface holes. Further targeted 2D grain characterisation by optical microscopy (OM), scanning electron microscopy (SEM), and energy dispersive X-ray spectroscopy (SEM–EDS), following an intermediate manual correlative preparation step, revealed Fe-rich nodules within the tunnels. Finally, nanotomographic imaging by focussed ion beam microscopy (FIB-SEM) revealed coccoid and filamentous-like structures within subsurface tunnels, as well as accumulations of Fe and S in grain surface crusts, which may represent a biological rock varnish/glaze. We attribute these features to biological processes. This work highlights the advantages and novelty of the correlative imaging approach, across scales, dimensions, and modes, to investigate biological weathering processes. Further, we demonstrate correlative microscopy as a means of identifying fingerprints of biological communities, which could be used in the geologic rock record and on extra-terrestrial bodies.

## Introduction

Colonization of the land by plants and other organisms during the early Palaeozoic (~ 500 Ma^1^) was fundamental to the evolution of terrestrial landscapes. The expansion of primordial vegetation had an influential effect on the architecture and evolution of river and sedimentary systems^2,3^, weathering and soil development^4–6^, and crucially the drawdown of atmospheric CO_2_ through organic carbon burial and weathering^7,8^. The first plant-dominated biospheres were akin to modern cryptogamic ground covers (CGCs)^4,9,10^, which are composed of a consortia of early divergent and evolutionary ancient non-vascular bryophyte plants (mosses, liverworts, hornworts), lichens, fungi, algae, and bacteria. At the modern day, CGCs are present in a variety of habitats ranging from deserts to polar tundra^11–13^, and often are the pioneering organisms of bare land surfaces before the vascular plants. Modern CGCs are considered suitable analogues for early terrestrial biotas because of the similarity between the modern and ancient plant components (cryptophytes) and the relationships that they developed with other organisms^4,10,14^. Importantly, symbioses between plants and fungi (e.g. mycorrhizae) and between fungi and algae or cyanobacteria (e.g. lichens) were also present during the early Palaeozoic, with the exceptionally preserved 407 million year old Rhynie chert biota providing many examples^15–17^. It is generally regarded that symbionts in the early Palaeozoic were responsible for a portion of the biologically mediated weathering via targeted nutrient (elemental) acquisition from soil minerals^18,19^, followed by development of the first biologically-mediated ‘proto-soils’^13^ and eventual global influence on biogeochemical cycles^19,20^. However, the mechanisms of weathering in both the modern and ancient examples are poorly understood. Investigating the intricate nano-to-micro scale interactions in modern analogous CGCs can shed light on how ancestors of these primitive organisms contributed to soil-forming processes, biologically mediated weathering, and potential nutrient-acquisition from their substrates.

Previous research on modern CGCs developing on primordial land surfaces from Iceland (i.e. lava flows and volcanic sedimentary terrains that are relatively new and unweathered land surfaces devoid of ‘higher’ vascular plants) were mostly limited to 2D investigations, mainly of grain surface processes^21^. These revealed biologically-mediated weathering features (BWFs) on volcanic glass and scoria within soils that were attributed to the actions of different organisms (e.g. bacteria, fungi) and processes (e.g. symbiosis, root-mediated dissolution)^21^. Prominent among these were ‘tunnels’, which are thought to be created by ‘boring’ fungal hyphae^21–32^. These tunnels differ from holes that develop naturally as gas-escape vesicles in volcanic ejecta because they generally form elongate tubes, are associated with organic material, and are not present in every grain, and the tunnels are generally much smaller than the vesicles. Here, we develop a novel correlative microscopy workflow across modes, dimensions and scales to investigate the physical, chemical and morphological characteristics of these tunnels. Our approach combines optical microscopy (OM), scanning electron microscopy (SEM) imaging and chemical analysis (SEM–EDS), high resolution X-ray microscopy (tomography) (XRM) and focussed ion beam microscopy (FIB-SEM), that are correlated using ZEISS ZEN Connect and Atlas 5 (3D) software. Correlative microscopy has the advantage that numerous data types can be acquired and studied in-situ, in unison, and across dimensions, therefore providing a holistic approach, and enabling a better understanding of how different parameters (e.g. morphology, chemistry, structure) are linked. This approach is currently a developing application in human-made materials research^33–36^ and life/biological science^37–40^, and despite a few geological^41–43^ and more recently specific soil science examples^44,45^, the method has not been applied to soil weathering and biological interactions.

We adopt the following correlative strategy. First, we document the tunnel networks through high resolution 3D XRM, which provides insights into their 3D morphology and how they might have formed. Second, we show how 3D tomography can be correlated with high resolution imaging and chemical data derived from SEM–EDS to provide information relating to the tunnel elemental variations and micro-to-nano scale features. Finally, we utilise the correlative microscopy workflow to target specific regions of interest for further analysis via FIB-SEM and generate nanotomographic volumes, which not only increases resolution (small pixel/voxel sizes) but also provides complementary nested 3D information to XRM. Our aim is to use this correlative approach to characterize weathering, potentially of a biological origin, in modern analogues of early land-plant communities. Our ultimate goal is to develop the use of features on the micro-to-nanometre scale as fingerprints of biological community presence and indicators of biologically mediated weathering in the geologic past. Further, we propose that this approach could be used in the search for biological influences on extra-terrestrial bodies.

## Results

### 3D multi-scale imaging of soil core, subsurface segmentation, and grain (digital) isolation

**Figure 1:**
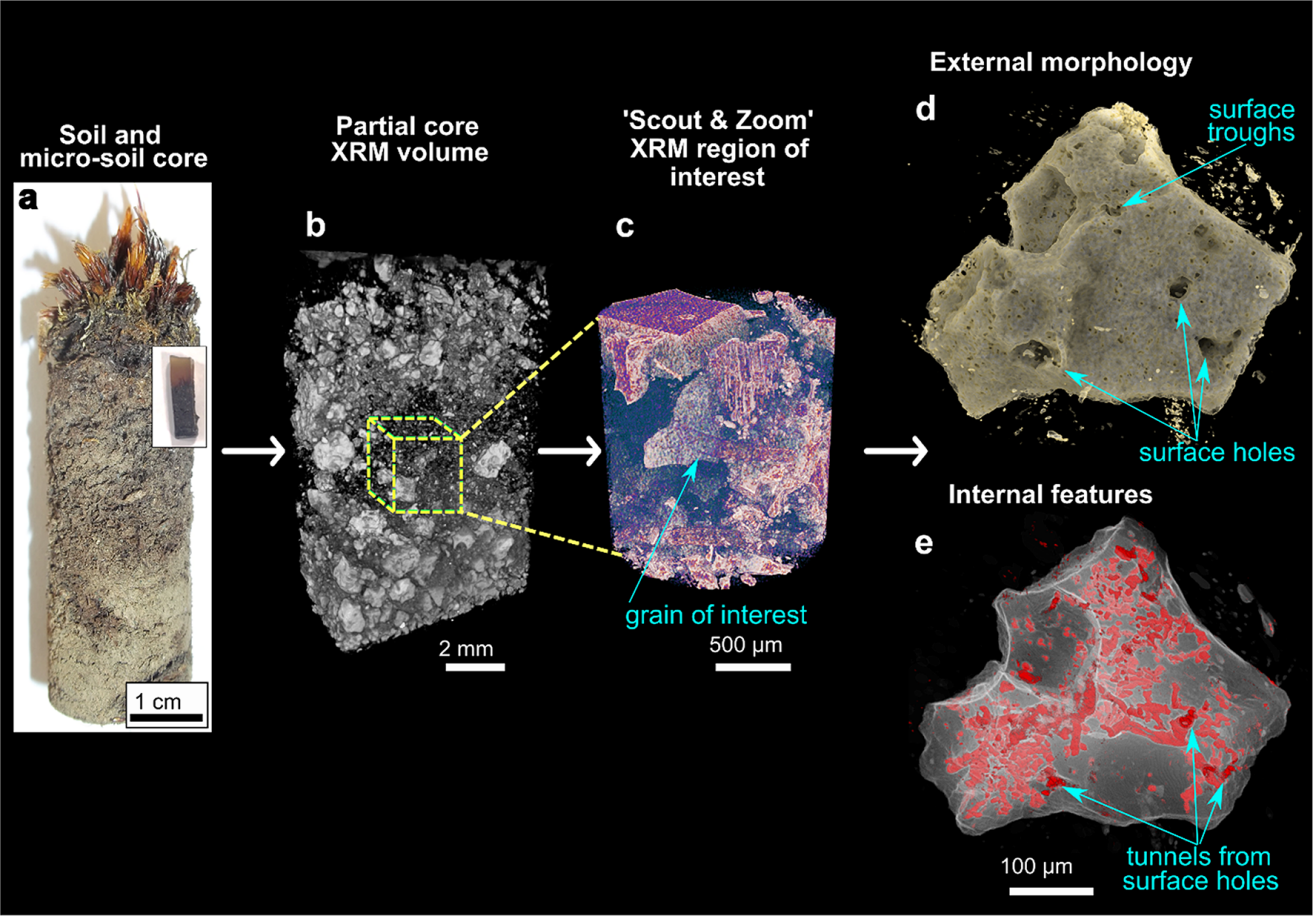
The correlative 3D imaging process: illustrates progressive higher resolution study from whole CGC soil core **(a),** to micro-core, Scout and Zoom feature on ZEISS Xradia Versa 520 **(b, c),** and finally segmentation of grain tunnels **(d, e)** (also see Supplementary Videos S1-3).

**Figure 2:**
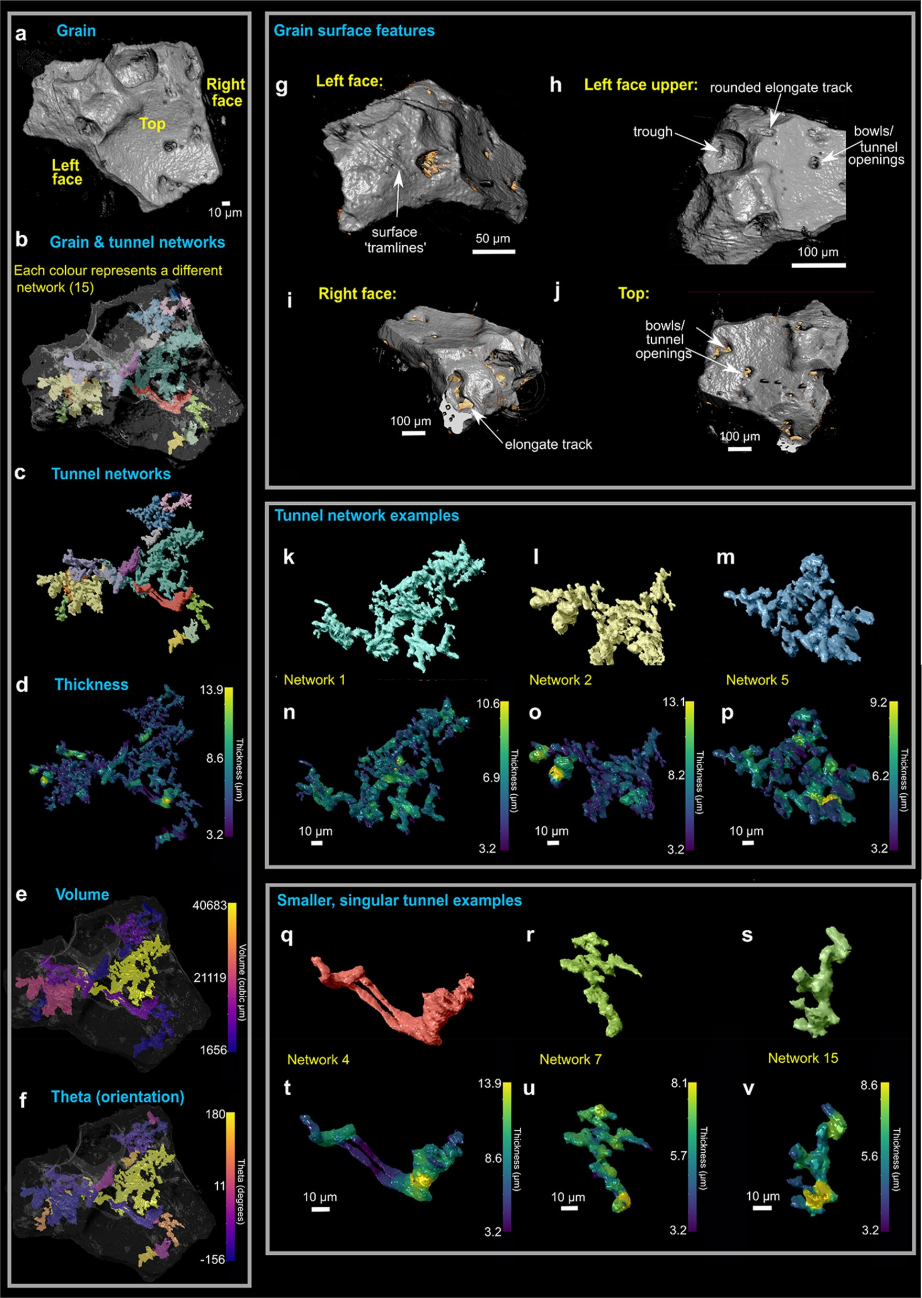
3D analysis of the segmented tunnel networks from the grain of interest. **(a-f)** The entire grain tunnel networks including each individual network segmented as its own colour **(a, b),** tunnel thickness variations **(d)**, volume variations **(e)**, and orientation variations **(f)**. Grain surface features also shown **(g-j);** features include surface holes, tramlines, troughs, bowls, and rounded elongate tracks. Gold colour indicates accumulation of organic material. From analysis, there appears to be larger tunnel networks **(k-p)** and those that are more singular **(q–v)**; variations in thickness through the tunnels are shown. Also see Supplementary Video S3.

3D tomographic imaging of a CGC soil micro-core was achieved using the ZEISS Scout-and-Zoom workflow on a ZEISS Xradia 520 Versa XRM (Fig. 1). From initial whole-core scans (Fig. 1a, b), progressively higher magnification and resolution (i.e. smaller voxel sizes) in subsequent scans (Fig. 1c) enabled us to identify, target and image a ~ 300 µm diameter in-situ volcanic scoria grain from the central subsurface region of the micro-soil core (Supplementary Videos S1-2). The scans revealed that the grain has both surficial and internal features of interest visible to a voxel size of less than 1 µm (Fig. 1d, e, Supplementary Videos S3-4). Following segmentation of the grain and its internal features, these resolved into a) networks of branched and sometimes interconnected tunnels of varying morphologies and characteristics (Figs. 1e, 2a–f, k–v, Supplementary Video S3), and b) a series of holes and troughs on the surface of the grain (Figs. 1d, 2g–j).

The grain surface features are diverse, representing holes, troughs, and elongate tracks of different orientations, lengths and shapes (Figs. 2g–j). The tunnels appear to originate from holes at the grain surface (Figs. 1, 2a, g–j), which extend to varying depths within the grain, and also appear to contain accumulations of organic material (Figs. 2g–j), but these are at the limit of resolution of Versa XRM. The individual tunnel networks were segmented for volumetric and morphological analyses, and were provided with a specific colour for ease of locating them within the grain (Figs. 2a–c). 3D segmentation reveals that the tunnels make up 20% of the grain. The tunnel networks appear to fall into two morphological groups: those that are branched (e.g. tunnel networks 1, 2 and 5; Figs. 2 k–p) and those that form singular, closed channels (e.g. tunnel networks 4, 7 and 15; Figs. 2q–v). Performing morphological analysis from the XRM segmentations reveals that all tunnels range in thickness from 3.2 to 13.9 µm (Fig. 2d) and the most voluminous are in the largest, most networked tunnels (e.g. networks 1 and 2; Figs. 2e). The thickness varies throughout each of the networks, however the thickest portion is usually at the entrance/exit hole (Figs. 2k–v). Networks also don’t have a particular orientation in the grain and are varied across 360° (Fig. 2f). Thus the tunnel networks can be characterised by their shape, morphology, and the way that they branch.

### Correlation of 2D and 3D datasets and correlative preparation step

**Figure 3:**
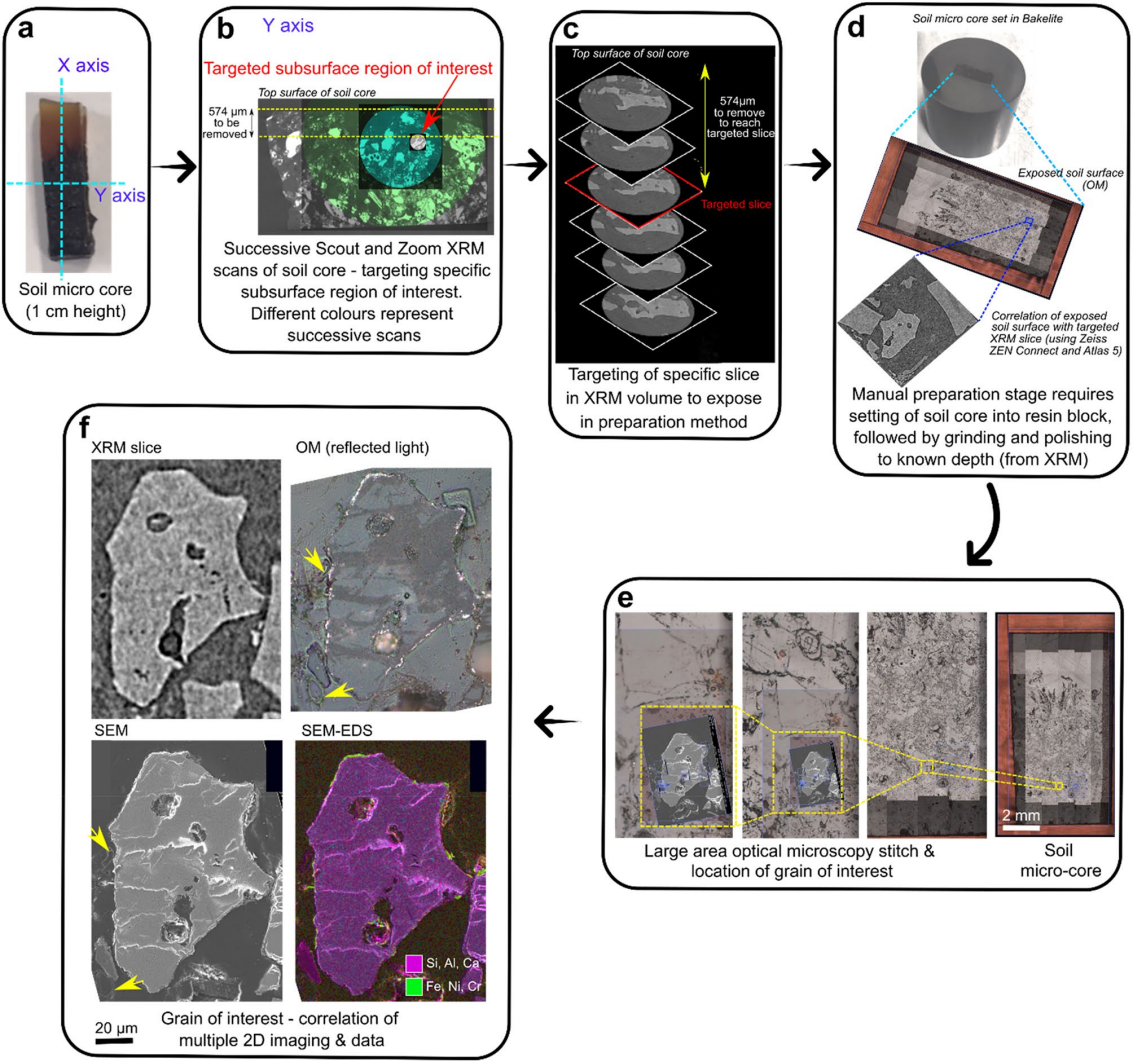
Additional correlative sample preparation step, revealing grain and slice of interest for further study via other imaging and chemical methods. **(a)** Axis orientations of soil micro-core. **(b)** Progressively higher resolution 3D volumes obtained from XRM are correlated, focussing on grain of interest (red arrow). **(c)** An assessment of depth of material to be removed (and from which axis) determined from XRM scans. Targeted slice from XRM scans at 574 µm depth. **(d)** Soil micro-core mounted in bakelite, and manually ground and polished to remove specific depth of material (574 µm); measurements taken regularly using a calliper (see methods section). **(e)** Large area stitch imaging was completed via optical microscopy to image the grain of interest to high resolution in 2D. **(f)** Subsequently the grain of interest underwent numerous 2D imaging and analysis methods including SEM, OM, SEM–EDS, and correlation with the XRM slice. Yellow arrow indicates plant material surrounding the grain within the soil matrix.

The correlative imaging workflow enables the combination of 3D and 2D datasets from multiple modes of acquisition. By using advanced correlative software (ZEISS ZEN Connect and Atlas 5 (3D)) it is possible to target specific subsurface regions or features of interest from the 3D XRM data and expose it through a separate correlative preparation step (Figs. 3a–d), allowing further targeted study in 2D (i.e. through SEM imaging, SEM–EDS chemical mapping, FIB-SEM, or other techniques not used here; Figs. 3e–f). Subsequent imaging via OM and SEM of the same region is overlaid using ZEISS ZEN Connect and combined with 3D XRM data (Fig. 3d, Supplementary Video S5).

### 2D imaging of newly exposed grain surface (OM and SEM)

**Figure 4:**
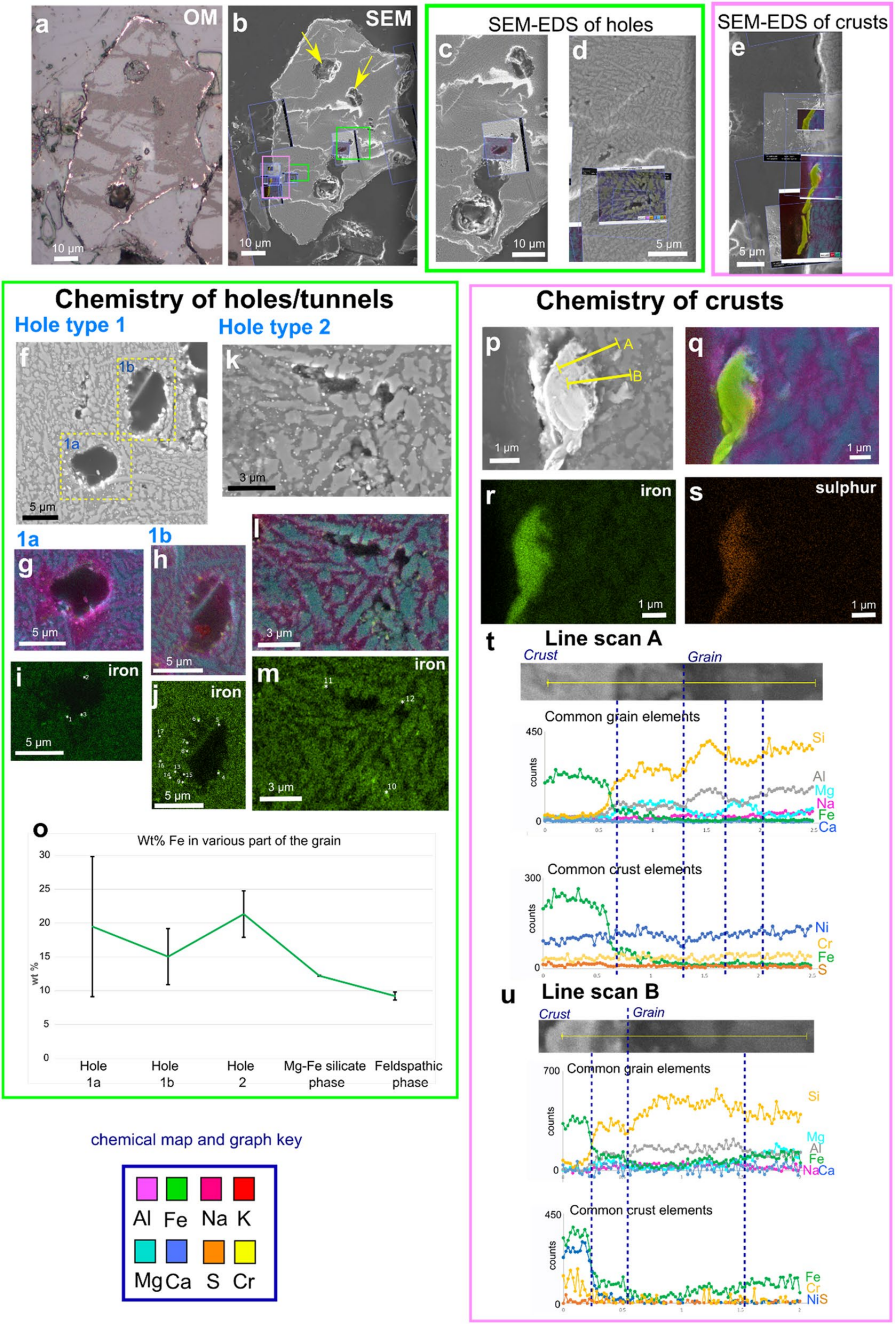
**(a, b)** Correlation of 2D imaging (OM, SEM) views of exposed grain of interest. **(b)** location of targeted areas of interest for SEM imaging and analysis via SEM–EDS. These were collected ‘live’ to enable correlation to specific areas (Supplementary Video S5). Yellow arrows indicate vesicular gas escape structures. Green box indicates holes of interest **(c, d),** while pink box represents grain surface crusts **(e**). **(f-m)** SEM imaging and SEM–EDS maps of tunnel cross sections (holes) from exposed surface. Two types of hole are identified. Chemical and morphological variations in grain mineralogical phases also shown. **(o)** Wt% of Fe variations shown for different hole types and the different mineralogical phases; spot analyses locations shown in **(i-m)**. **(p-s)** SEM imaging and chemical SEM–EDS maps of grain surface crusts shown; chemical line scans in **(p)** shown in **(t-u).** Y axis in **(t-u)** is counts per second. The brighter grey mineralogical phase contains Mg, Ca and Fe (interpreted as a Mg-Fe silicate phase), and a darker grey mineralogical phase containing Al, Na, K, Si and O (interpreted as a feldspathic phase). SEM images collected using SESI detector.

SEM and OM imaging of the newly exposed volcanic glass grain surface reveals a pseudosymplectite texture (Fig. 4); pseudo refers to the phases in this volcanic glass grain which are not true minerals, and symplectite refers to a petrographic microstructure with intergrowths of two or more phases where one (or more) phase may be more unstable than the other(s), and recrystalises during formation to more stable constituents under changing pressures, temperatures, and/or interaction with external fluids^46^. Chemical analysis of the grain via SEM–EDS indicates that there are two chemically distinct psuedo-mineralogical phases in the symplectite: a brighter grey phase (from SEM greyscale imaging) containing Mg, Ca, Fe and Si (interpreted as a Mg-Fe silicate phase), and a darker grey phase (from SEM greyscale imaging) containing Al, Na, K, Si and O (interpreted as a feldspathic phase) (e.g. Figures 4f-m, p-q). The grain contains large gas-escape vesicles (Fig. 4b) as well as the smaller tunnel networks; the vesicles were omitted from the XRM segmentation process.

2D cross sections through tunnels of two different morphologies were investigated further, hereafter named hole types 1 and 2 (Figs. 4f–o). In hole type 1, sections reveal tunnels with a circular to elliptical outline 5–10 µm in diameter (Figs. 4f, g–j). Results show that these tunnels cut across the boundaries of the feldspathic and Mg-Fe silicate phases. The tunnel outline is smooth and curved, where the rim is bright in the SEM images, which appears to be due to the accumulation of heavier elements (Fe; Figs. 4g–i).

These accumulations appear nodulous and are often accompanied by high S but not always (Supplementary Data S6). SEM–EDS spot analyses of the nodules indicate that Fe concentrations range between 11 and 32 wt%, S is up to 1 wt%, and the average for hole type 1 is between 15 and 20 wt%. The hole type 2 (Figs. 4k–o) morphologies are irregularly shaped being more elongate and have a diameter of ~ 1–2 µm. They formed entirely within the feldspathic (Al, Na, K, Si) phases leaving the Mg-Fe silicate phase intact (Figs. 4k–m). As with hole type 1, they contain Fe-rich nodular accumulations on some hole edges (Figs. 4l–m). SEM–EDS spot analysis indicates the nodules have Fe concentrations ranging between 17 and 25 wt%, and negligible amounts of S (Supplementary Data S6). The average chemical compositions of the Fe nodules in both hole types is higher than the Mg-Fe silicate phase of the grain, with a larger variability particularly in hole 1a (Fig. 4o).

Reflected OM imaging indicates that there are bright regions on the outside edge of the grain (Fig. 4a) forming irregular and non-continuous crusts. Further investigation via SEM indicates that the crusts vary in morphology, generally forming 1–2 µm thick surface coatings that are not continuous over the entire grain edge. Some crusts also appear as coatings within the larger gas escape vesicles inside the grain (Fig. 4a). The boundary between the crust and the grain surface is sometimes abrupt, but often gradational, developing a mixed, transition layer (Fig. 4e, p). SEM–EDS analysis indicates that the crusts are an accumulation of heavier elements including Fe and S, where Fe is again in higher proportions compared with the ‘background’ Mg-Fe silicate phases of the grain (Fig. 4r). SEM–EDS line scans across the crust-grain boundary indicate abrupt chemical changes, particularly in Fe and Si, although low counts for Si are still collected in the crust. The transition layer appears to form an intermediate zone of mixed chemistry (Figs. 4t, u).

### Nanotomography of tunnel and crust morphology from targeted FIB-SEM milling

**Figure 5:**
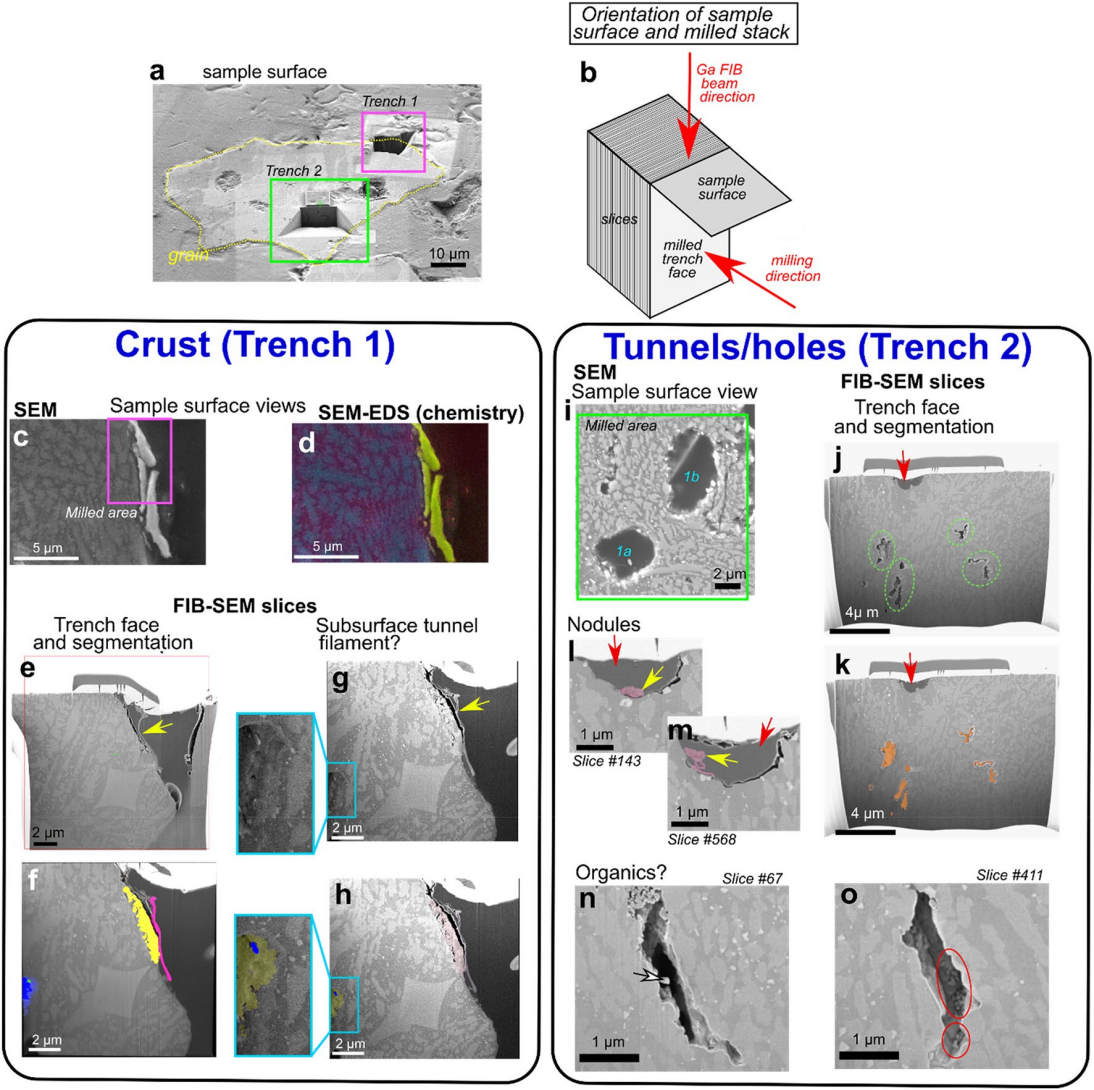
Location of FIB-SEM trenches and tomographic volumes. **(a)** Exposed grain from targeted XRM slice and surface material removal. Blue boxes highlight the milled trenches (1 and 2) **(b)** Schematic explaining the orientations associated with trench milling and sample surface. **(c-h)** Trench 1 (crust). Sample surface view **(c)**, accompanying chemical map (key the same as for Fig. 4) **(d),** and view of the trench face after Atlas 5(3D) sample preparation and fine polish **(e–h);** yellow arrow indicates filamentous structure on crust. **(f, h)** Trench face highlighting false colour segmented components; yellow = crust, pink = filamentous portion of crust, blue = interior tunnel, green = (probable organic) filament within tunnel. **(g)** Final post-mill trench face highlighting subsurface tunnel containing a filament, with segmented version **(h). (i-o)** Trench 2 (tunnels/holes). **(i)** Sample surface view showing the milled area over hole types 1a,b from Fig. 4. **(j, k)** Trench face highlighting subsurface tunnels that are unobservable from XRM imaging (green circles) and surface holes (red arrow). Segmented subsurface tunnels shown in **(k). (l, m)** Close up view of surface holes (red arrows) from two different slices through the volume highlighting segmented Fe nodules (yellow arrows). **(n, o)** Two examples of slices of subsurface tunnels, both exhibiting inhabiting potential organic filamentous (white arrow) and coccoid structures (red circle).

**Figure 6:**
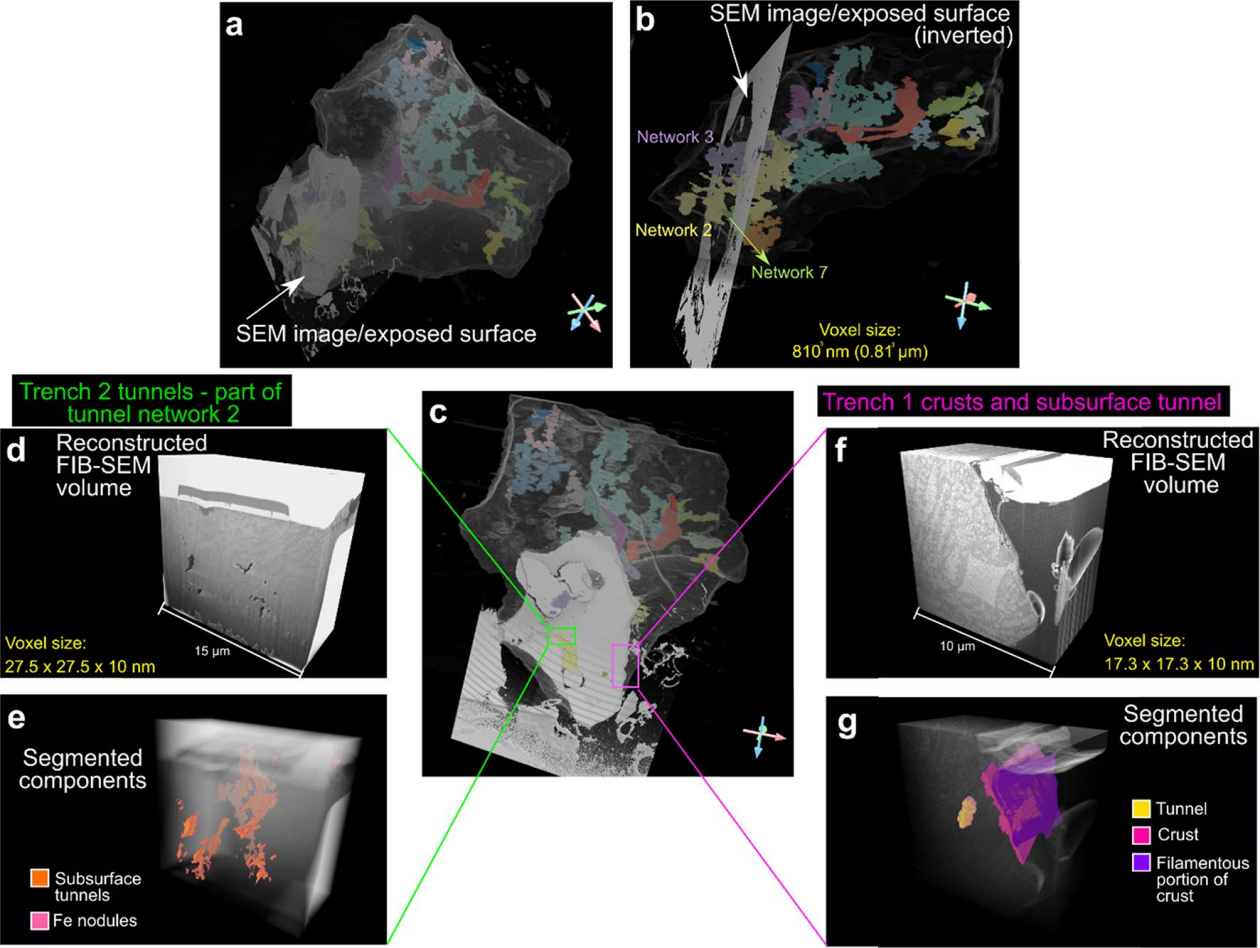
3D XRM grain with segmented tunnels correlated with SEM image of exposed grain and location of FIB-SEM volumes. **(a)** Location of SEM image/exposed surface in relation to the entire grain, and which segmented tunnel networks interact with it (networks 2, 3 and 7) **(b)**. **(c)** Location of FIB-SEM volumes for trench 2 (tunnels/holes) **(d, e)** and trench 1 (crusts) **(f, g).** Trench 2 tunnels are identified as belonging to tunnel network 2 in Fig. 2.

High resolution 3D volumes of the tunnels and the surface crusts were obtained through targeted FIB-SEM nanotomographic milling (Figs. 5, 6). This not only complements the XRM imaging and data, but also enables further study of crust structure through combined higher resolution imaging and element analysis when XRM resolution limits are reached. Two trenches were destructively milled away using the Ga FIB beam, the locations and orientations of which are illustrated in Figs. 5a, b; the scanning parameters of each can be found in Supplementary Methods S7. Milling and subsequent segmentation of a grain surface crust in trench 1 indicates an isolated subsurface crust of a different morphology to the rest of the grain, and a curious filamentous fragment appressed to the crust surface (Figs. 5e–h). Additionally, a subsurface tunnel is observed which appears to contain a filamentous fragment (Figs. 5g–h, box inset and enlargement). Because of the limited size of the milled volume, the extent of the subsurface tunnel through the rest of the grain is unclear.

Surface troughs and subsurface tunnels are also identified in milled trench 2 of tunnel/holes cross sections (Figs. 5i–o). The holes are infilled with embedding resin (Figs. 5j–k, red arrows) and have smooth and rounded sides (like their counterparts in Figs. 4f–j). Subsurface tunnels are again identified; these are more elongated, show greater irregularity, and appear to be located in both feldspathic and Mg-Fe silicate phases (Figs. 5j–o, 6d–e). Surface holes contain irregularly shaped nodular objects with some filamentous structures (Figs. 5l, m, yellow arrows). Finally, some subsurface tunnels seem to contain filamentous and coccoid structures (Figs. 5n, o, 6). 3D volumes of each FIB-SEM stack can be seen in Fig. 6 and Supplementary Videos S8, 9.

## Discussion

**Figure 7:**
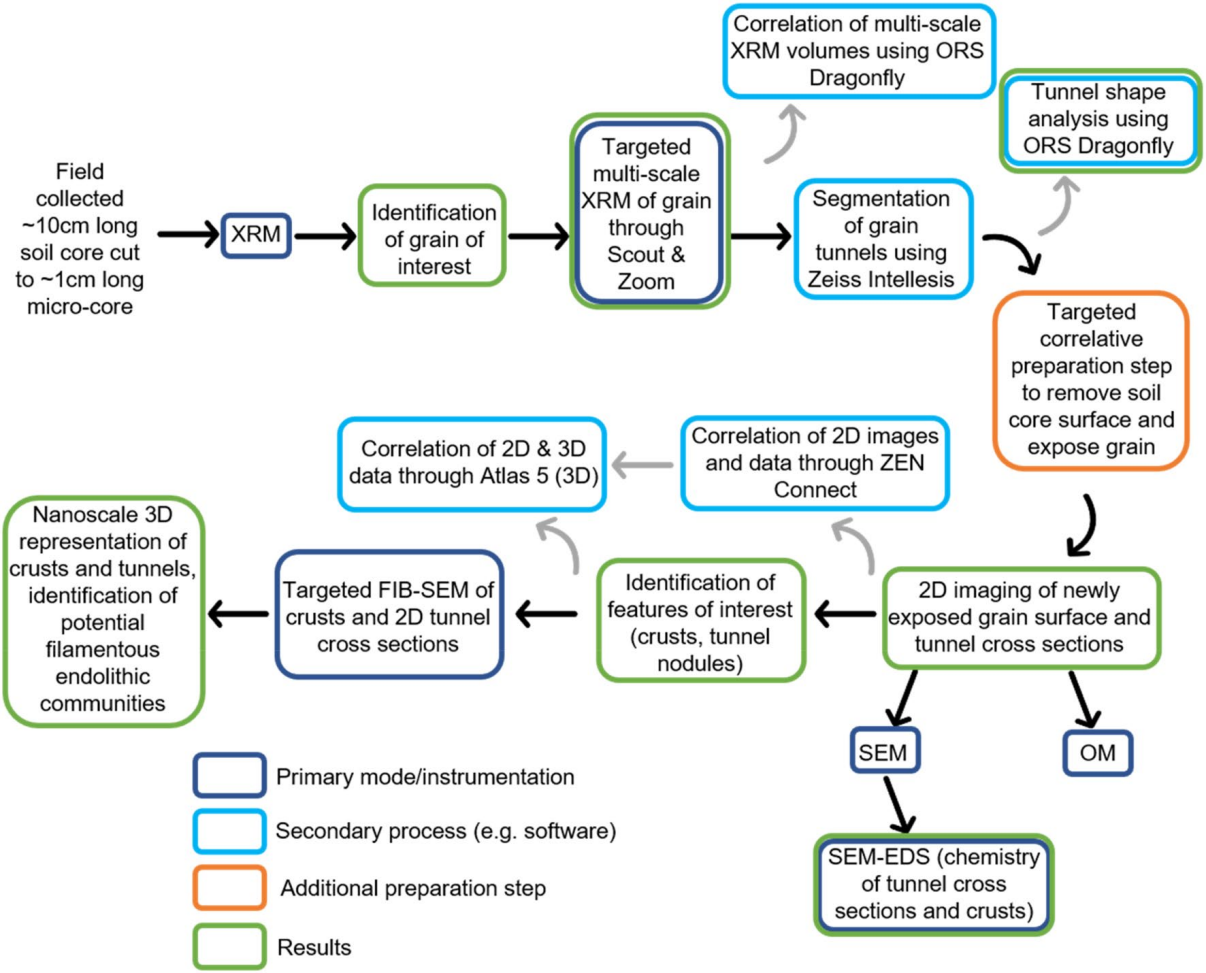
Flowchart summarising the targeted correlative workflow employed in this study.

Our results demonstrate that multi-modal correlative microscopy provides a novel method for understanding the multi-scale processes involved in soil weathering, specifically when these processes (e.g. tunnel formation) occur in three dimensions. The correlative approach is becoming increasingly used across the materials^36^ and biological^38^ sciences and has distinct advantages over conventional ‘single mode’ approaches. The correlative workflow overcomes the restriction of studying in one scale/dimension/technique alone by combining (layering) imaging and other data (e.g., chemical, crystallographic), while also successively improving resolution (Fig. 7, Supplementary Videos S1, 2, 5); for example, FIB-SEM pixel (voxel) sizes are vastly smaller than those obtained from conventional laboratory XRM instruments (17.3 nm vs 0.81 µm for our results, respectively; Figs. 6b and f, Supplementary Methods S7, 10), allowing complementary analysis by bridging micro-to-nano scale features with reciprocal context and improving information output (Fig. 6). Studying objects across dimensions and scales also reveals characteristics and features which might not otherwise be identified via a single technique or in one dimension alone (e.g. the morphology of tunnel networks and the presence of grain crusts). Finally, the ability to target specific subsurface regions of the soil grain of interest within a core sample through initial ‘coarse’ non-destructive 3D XRM imaging (Supplementary Videos S1, 2), subsequent correlative preparation steps, and successive combined analytical and imaging approaches enabled the study of a specific object in the context of its microenvironment (i.e. the ‘targeted trajectory approach’ of^36^). Correlative imaging thus allows us to study the combined 2D and 3D morphological and chemical characteristics of cryptogamic ground cover soil and grain weathering.

Our findings demonstrate that an assortment of markings are present on the surface of a soil grain of interest. These are reminiscent of the surficial bowls, tramlines, elongate troughs, and internal pore networks previously described as biologically mediated weathering features (BWFs) by^21^ that are common in modern CGC soils. Although no microorganisms were observed colonising the grain surface from our XRM scans (the voxel size is not small enough to resolve them; Supplementary Methods S10), the presence of these features could suggest a biological origin. Indeed, accumulations of organic material are present within holes and troughs (Figs. 2g–i) and surrounding the grain in the soil matrix (Fig. 3f yellow arrows, Supplementary Videos S2, 4). This, to our knowledge, is the first description of 3D surficial grain BWFs associated with CGCs.

3D volumetric analysis of tunnels, high resolution 2D imaging with chemical analysis, and the correlation of datasets establishes that the holes/tunnel networks studied in Figs. 4f–m, 5i–o belong to tunnel network 2 (Figs. 6a–c). The variations in morphology between the two types of tunnel (i.e. shape and volume; Figs. 2k–v) signify that they might have been formed by different processes; either abiotic and/or biotic. One hypothesis could be the abiotic dissolution of easily weatherable mineral phases by acid rain, which is a common atmospheric feature following recent eruptions in Iceland^47^. How much this would affect proto-soil grains in CGCs though is unclear, and would be affected by the time the grains were under chemical attack, depth of grains within the soil, and their proximity to eruptions. A second hypothesis is that the tunnels formed through abiotic chemical dissolution where soil waters, potentially enriched in CO_2_, dissolved easily weatherable minerals. This could be exacerbated by below-ground biological respiration of CO_2_ and exudates from microbes could indirectly be responsible for mineral attack and dissolution^48^. 2D imaging (Fig. 4) reveals evidence of hole formation that follows specific localized grain chemistries (hole type 2; Figs. 4k–m) where preferential dissolution occurs in the feldspathic phase. This could indicate that these parts of the grain are more ‘easily weatherable’ and prone to chemical attack. Interestingly, evidence of biologically mediated feldspar weathering is common in the literature^29,30,49^; it is also reported that the presence of feldspars in rocks may increase the susceptibility for biological attack by fungi, and that the rock’s original chemistry and mineralogy highly influences these physical and biochemical effects^50,51^. This is likely because feldspars contain many of the essential elements (e.g. Ca, Na, K) needed for microorganisms and their symbiotic partners to live^30,52^. Therefore, a third hypothesis is that the tunnel formation is due to biological factors. Hole type 1 tunnel morphology cuts across chemical boundaries within the grain. This difference might be a factor of time, where hole type 2 morphologies are created first and a longer duration of weathering leads to the creation of larger, more rounded and branched tunnels (hole type 1). Hole type 1 has rounded sides and circular cross section compared with type 2; these are reminiscent of 2D tunnel structure observed in other studies^21,28^ which are reported as being from a biological origin, usually fungal^53^. Fungal tunnels within soil minerals have been explained as a result of dissolution and ‘boring’ by combined biomechanical forcing and biochemical alteration; the tunnel retains its shape following the death and degradation of the hyphae^27,49,50^. Other documented cases of fungal borings of mineral grains produce tunnels of variable form, including simple/straight, branched, helical/coiled and annulated^28,53^, often with constant diameters and rounded ends^4,54^, sometimes forming anastomosing ‘channels’^29^. There is a single 3D study within garnets describing tunnels as straight and funnel-shaped with rectangular cross sections becoming more rounded towards the tip^26^. In 2D, our results match closely with these morphological descriptions from the literature, however in 3D our tunnels are not uniform in shape or width (Fig. 2), being neither particularly straight nor funnel shaped, questioning whether they can be attributed to fungi, or indeed, to a biological origin. Grain surficial holes/bowls, which represent the openings of subsurface tunnels (Figs. 1, 2), contain accumulations of organic material (Figs. 2g–j) implying intimate connections to living organisms. The tunnels might have formed through chemical dissolution by bacterial communities rather than through biomechanical borings by fungal hyphae. If this is the case, the feldspathic phase likely weathered first, and the Fe–Mg silicate phase later, which creates the difference in tunnel morphologies. Fungal hyphae may have colonised pre-existing cracks or fissures in the grain^50^, the presence of which is supported by the identification of surface BWFs likely caused by both fungal hyphae and bacteria^21^. So although we cannot be certain what exactly was causing the tunnels, we have shown that by taking a correlative, multi-dimensional and multi-scale approach, we have the ability to study weathering features in a more holistic way than by one or two techniques alone.

FIB-SEM milling reveals potential communities of endolithic microbes evidenced by bacterial-like filaments and coccoid-like structures within tunnels (Figs. 5g–h, j–o), subsurface colonisation likely providing protection from environmental extremes^50^. Although we cannot be sure what these organisms are from FIB-SEM imaging alone, and the lack of evident internal structures, the shape and size suggest they are not fungal hyphae, but could be a mix of cyanobacteria-fungi-lichen biofilms, lichenised and non-lichenised fungi, and yeast-like unicellular fungi, which commonly form endolithic microbial communities^5,50,55,56^. These organisms might have enhanced other forms of biologically mediated weathering through the in-situ secretion of organic acids and other exudates, leading to the irregular (non-straight/funnel) shape of the tunnels. Their existence is further supported by the presence of Fe-rich nodules in both types of tunnel (Figs. 4g–m). Fe-rich nodules are thought to be indicators of fungal hyphae bio-precipitation in modern CGCs^21^, with further occurrences reportedly created by lichens^57,58^, bacteria^48^, other fungi^59–61^, and iron oxidising bacteria^62^. The Fe concentration of the nodules is higher than in the feldspathic and Mg-Fe silicate phases of the grain (Fig. 4o), indicating a separate source, which could be biologically derived. A biological source could also explain variations in Fe concentration observed in the nodules.

The imaging and analysis results presented here demonstrate that some surfaces of the grain of interest are covered in a crust of specific and distinctive chemical composition (high Fe and S; Figs. 4, 5). XRM scans show some brightening of the surface indicating the presence of higher density material (Fig. 3f). However, because the crust thickness (< 2 µm; Fig. 4) overlaps significantly with the voxel size resolution of the scans (0.81 µm), it cannot be conclusively segmented, which highlights the need to combine datasets from multiple modalities (and resolutions) through correlative microscopy. Our results demonstrate that crust morphology is variable, but because of their heavier element chemistry, they appear brighter in SEM and optical imaging, which is a phenomenon observed in other studies^63^. The crust chemical composition is distinctly different to the ‘normal’ composition of basaltic rocks and glass (Fig. 4), which indicates alternative modes of accumulation and formation. We discard the possibility that these crusts are due to contamination because a) there is evidence from the initial XRM scans, albeit at limited resolution, of bright areas on the outside of the grain; these precede any manual preparation, b) pristine grinding papers devoid of any contamination were used during the correlative preparation step, and c) the micro-soil core was already set in epoxy resin. One possible explanation could be the volcanic source of the grain. Nickel and chromium are common in early formed minerals during volcanic eruption, where nickel can be incorporated into the forsterite (Mg end member of olivine) chemical structure^64^. This however seems unlikely as the crusts are localised to grain surfaces and don’t appear to form internally. An alternative hypothesis could be that the patchy formation of the crust on the grain surface could be due to localised biological interactions. Various rock varnishes, coatings, weathering rinds and glazes are known^48,65,66^, some specifically caused by fungi^50,66^ and epilithic lichens^50^. It is well established that key chemical diagnostic features of biologically-mediated rock varnishes, glazes and coatings traditionally includes high accumulations of Mn and/or Fe^48,65,67,68^. Biomineralization of these elements as surface coatings, varnishes and glazes results from the oxidation/reduction of the metal, usually because of excretion of oxalate and/or hydroxycarboxylic acids by a variety of microbes including fungi and bacteria^48,50,58,69^. While the crusts outlined here do not have significant accumulation of Mn, suggesting that Mn oxidising and reducing bacteria could be absent from this CGC soil biosphere, they do have high Fe compared to the background grain chemistry (Fig. 4). Lichenised fungi are known to biomineralize Fe-rich minerals on basaltic lava flows^69^ and lichenised cyanobacteria can biomineralize Fe hydroxides and clay-coatings to develop rock varnishes^70,71^. Therefore, it could be that our crusts are produced by microbial bioprecipitation, potentially by lichenised fungi and/or bacteria. Cr in the crusts could also be due to fungi, which can precipitate reduced forms around their cells^69,72^. The presence and formation mechanism of these crusts could be via the same processes as the Fe-rich nodules (Fig. 4); the composition of Fe is similar, however the nodules appear to lack sufficient proportions of Cr and/or Ni. Although we cannot conclusively state that the crusts are formed from biological interactions, it provides a plausible hypothesis based on their morphology, chemical composition, the evidence for likely colonisation by fungi and cyanobacteria from grain surface BWFs (Figs. 2g–j), the subsurface weathering features, and potential endolithic communities (Figs. 1,2,4,5).

## Conclusions

This work highlights the advantages and novelty of using multi-scale and multi-dimensional correlative microscopy to understand weathering in cryptogamic ground covers (CGCs), allowing targeting of specific sub-surface soil regions for further study with complementary techniques. From targeted multi-scale X-ray Microscopy (XRM) imaging, we have identified numerous surficial grain features which are analogous to previously described biologically mediated weathering features (BWFs) and internal tunnels, which are also likely the products of biological weathering processes, whether directly from fungal borings or indirectly via mineral attack from microbial exudates. Two types of tunnel were identified: those that form branched networks, and those that are more linear and singular. Following exposure of a cross section of the grain of interest through an intermediate correlative preparation step, we used optical microscopy (OM), scanning electron microscopy (SEM), and element mapping (SEM–EDS) to characterise the morphology and chemical characteristics of the tunnels. Results revealed micron-scale variations in morphology between the two types of tunnel and Fe-rich nodules within, which were probably formed through biological processes. Grain surfaces crusts were also identified. These have accumulations and variations in heavier elements (Fe, S), and could represent a type of biological rock varnish/glaze. Further focused ion beam (FIB-SEM) nanotomographic imaging of both tunnels and crusts not only improved resolution (voxel sizes) of small-scale features, but also revealed the presence of probable biological filaments and coccoid-like structures within tunnels. The presence of (a) grain surface BWFs, (b) Fe rich probable bioprecipitates, and (c) bacterial-like coccoid and filamentous forms within tunnels indicates that biology played an important role in the alteration and weathering of the grain. The physical and chemical features outlined here could be used as bioindicators to identify biologically mediated weathering in the rock record, and potentially on extra-terrestrial bodies. There is a particular need for this to study the interactions between Earth’s earliest terrestrial biospheres and their substrates through the Proterozoic to the earliest Palaeozoic, particularly because of the disparity of the timing of terrestrialisation between molecular, phylogenetic, and fossil information. Further studies should aim to quantify the biological interaction with their substrates (in particular, soil grains) in real time and in multiple dimensions to better understand biological weathering and the impact of micro-to-nano scale biogeochemical processes on Earth-scale biogeochemical cycles.

## Methods

### Fieldwork and soil core collection

CGCs were collected from various localities in Iceland, the core from this study sampled from 65 47.688’N, 16 46.384’W (location L1 in^4^). This core contained a mix of organisms including mosses (*Racomitrum sp., Ceratodon purpureus, Pohlia sp., Polytrichum juniperinum)* and unidentified lichens. An extensive description of the field site can be found in^21^. The core was cut and mounted in epoxy resin for thin section preparation; following this the main soil core was cut down to ~ 1 cm length to enable ease of mounting (and improved resolution) in the XRM.

### X-ray microscopy (XRM)

Micro-soil cores were scanned using a ZEISS Xradia 520 Versa X-ray microscope (XRM) for 3D tomography. The soil micro-core was attached using a cyanoacrylate-based adhesive to the end of a ~ 2 cm long pin and mounted onto a ZEISS specimen holder for scanning. Four scans were collected at various magnifications and fields of view to utilise the Scout and Zoom feature of the scanner (see Supplementary Methods S10); the final ‘high resolution’ scan being collected using the phase-enhanced contrast method. The ‘Scout and Zoom’ feature enables multi-scale study within the same regions of interest, enabling simple correlation of data at different scales (Supplementary Videos S1, 2). Reconstructed .txm files were converted to 8bit greyscale 2D .tiff image stacks. Initially, tunnels were identified in a grain of interest from a 2D .tiff stack (Supplementary Video S4), which was subsequently segmented to reconstruct the pore structure in 3D (Supplementary Video S3). Segmentation of tunnels was accomplished via the ZEISS ZEN Intellesis machine learning module within ZEISS ZEN Blue software v. 2.6; a number of slices from the imaged volume (in this case, 6) were manually ‘coloured in’ to reveal the different components within the scan (i.e. tunnels, grain, air) which was then applied to the rest of the volume for segmentation. Visualisation and quantification of tunnel thickness, volume and theta was achieved using Object Research Systems (ORS) Dragonfly software v. 2020.1. XRM scans and Intellesis segmentation were undertaken within the Advanced Imaging of Materials (AIM) Facility at Swansea University, UK, and ORS Dragonfly visualisation occurred within the Sheffield Tomography Centre (STC) at the University of Sheffield, UK.

### Intermediate correlative microscopy sample preparation step

Following the identification of a subsurface object of interest (in our case, the weathered grain) preparations can be made to expose the object for further study via a preparation stage. In this step, a ‘targeted slice’ was chosen from the XRM data, and from that a known amount of sample surface material (measured using the XRM scan images) can be removed. This is achieved via grinding and polishing of the sample surface^73^, and in our case, 574 µm (Fig. 3) needed to be removed to expose the targeted region. For ease of material removal, the soil micro-core was mounted in conductive Bakelite using an ATM Opal 410 mounting press, and subsequently ground and polished using sequentially finer grinding papers (320–600–1200–4000 grit). The resin block was frequently measured using a Hilka digital calliper to ensure the correct thickness of sample was removed. Further details on this manual correlative sample preparation method can be found in^73^. Our results were within 20 µm of the targeted slice (i.e., 554 µm was removed), so a new XRM slice was chosen for the following image correlation to match the newly exposed surface (Fig. 3); this 20 µm variation was likely due to the limited resolution of the digital calliper (10 µm). The newly exposed grain surface, still set in the Bakelite resin block, was coated with ~ 10 nm thickness of carbon using an Agar Scientific coater (Cressington, UK) for subsequent SEM imaging and analysis. This process was undertaken within the Advanced Imaging of Materials (AIM) Facility at Swansea University, UK.

### Correlative microscopy–correlating datasets

3D XRM data was initially loaded into ZEISS Atlas 5 (3D) software v. 5.2.1 installed on the ZEISS Crossbeam 550 FIB-SEM within the Advanced Imaging of Materials (AIM) Facility at Swansea University (UK). From there, once the specific amount of sample surface material had been removed to expose the object of interest via the correlative preparation step, imaging and data derived from SEM, SEM–EDS and OM were correlated manually using ZEISS ZEN Blue software v. 2.6 and the ZEISS ZEN Connect module, enabling a variety of datatypes to be overlain (Supplementary Video S5). From this, targeted FIB-SEM study was carried out utilising the combined 2D/3D approach in ZEISS Atlas 5 (3D) correlative software.

### Optical light microscopy (OM), Scanning Electron Microscopy (SEM) and energy dispersive X-ray spectroscopy (EDS)

Optical microscopy (OM) imaging was undertaken on a ZEISS Observer Z1M inverted microscope using ZEISS ZEN Blue software v. 2.6 with ZEN Connect. SEM images and SEM–EDS chemical analysis were undertaken on a ZEISS Crossbeam 550 FIB-SEM using Oxford Instruments X-MaxN 50 and Aztec software. A table illustrating the SEM imaging and data collection modes/analytical set up can be found in Supplementary Methods S7. Imaging and analysis occurred within the Advanced Imaging of Materials (AIM) Facility at Swansea University (UK).

### Focussed ion beam scanning electron microscopy (FIB-SEM)

Nanotomographic volumes were collected for subsurface tunnels and surface crusts using a ZEISS Crossbeam 550 gallium (Ga) source focussed ion beam scanning electron microscope (FIB-SEM) and Atlas 5 (3D) correlative software v. 5.2.1. The sample preparation for nanotomographic milling is as follows: After achieving an eucentric tilt correction, the sample stage is tilted to 54° so it is perpendicular to the FIB column. The sample surface is lifted with the stage vertical axis to 5 mm working distance where the two columns are in alignment, and then fine-tuned to confirm the FIB and SEM beams are at a coincidence point. Once a region of interest (ROI) is set using the overlay function of the Atlas 5 (3D) software (in our cases 15 × 15 µm, and 10 × 7 µm; Figs. 5c–o), setup can begin for the nanotomographic milling run. Firstly, an initial platinum layer is deposited on the overlay area using a gas injection system and the 30 kV 700 pA FIB probe. This protects the sample surface from damage by the Ga FIB beam and helps to create a cleaner cross section. 3D tracking marks, which facilitate automatic alignment, focus, astigmatism and drift correction as well as slice thickness tracking during the run, are milled onto the platinum layer using the 30 kV 50 pA FIB probe. These tracking marks are then infilled using the carbon gas injection system to provide enough contrast between the platinum and the tracking marks. A trench is then milled using the 30 kV 7nA FIB probe to produce a cross sectional face to a depth of approximately 15 µm. Finally, a lower energy probe is applied to the cross-sectional face using the 30 kV 700 pA FIB probe for more precision and lower interaction volume. The cross-sectional face is subsequently repeatedly milled (using the 30 kV 700 pA FIB probe) and imaged (using the SEM SESI detector with 1.8 kV pA beam) to create individual images (or slices; 10 nm thick) which can later be reconstructed into a 3D volume. Voxel sizes for each run include 17.3 × 17.3 × 10 nm for trench 1, and 27.5 × 27.5 × 10 nm for trench 2. Further image collection parameters can be found in Supplementary Methods S7. After a run of ~ 10 h, the image stack is aligned using the Fiji/ImageJ plugin StackReg^74^ and cropped in three dimensions using the Fiji/ImageJ plugin Crop3D to remove unwanted redeposition occasionally occurring on the edges of the imaged area. All 3D volumes (Figs. 5, 6) were visualised/rendered in ORS Dragonfly v. 2020.1. All of the above methods were conducted within the Advanced Imaging of Materials (AIM) Facility at Swansea University, UK.

## Data availability statement

Data is available in the supplemental materials.

## Supplementary Information

Supplementary Information 1.

Supplementary Information 2.

Supplementary Information 3.

Supplementary Video 1.

Supplementary Video 2.

Supplementary Video 3.

Supplementary Video 4.

Supplementary Video 5.

Supplementary Video 6.

Supplementary Information 4.
