# Supplementary material for: Correlative Microscopy: a tool for understanding soil weathering in modern analogues of early terrestrial biospheres
Source: Sci Rep. 2021 Jun 17;11:12736. doi: 10.1038/s41598-021-92184-1 (PMC8211647; doi:10.1038/s41598-021-92184-1)
Supplement: Supplementary file 3 — Supplementary Information 3. [file 41598_2021_92184_MOESM3_ESM.docx]

**Supplementary Methods 1 – Image and analytical acquisition set up for SEM, SEM-EDS, and FIB-SEM**

**Parameters for SEM imaging and SEM-EDS chemistry acquisition**

**SEM imaging**

Using Zeiss SmartSEM software

- Working distance: 5 mm
- Signal: SESI
- 10 kV
- Current: 1 nA

**SEM-EDS chemical analysis – chemical maps**

Using Oxford Instruments Aztec software

- Working Distance: 4.9 mm
- 15 kV
- Number of completed frames: 272
- Total counts: Range, but up to 48 million
- Process Time: 6
- Live time: up to 5348 seconds

**SEM-EDS chemical analysis – chemical line-scans**

Using Oxford Instruments Aztec software

- Working Distance: 5 mm
- 15 kV
- Number of passes: 10
- Total counts: Range, but up to 1 million
- Process Time: 6
- Live time: up to 1000 seconds

**SEM-EDS chemical analysis – spot analyses**

Using Oxford Instruments Aztec software

- Working Distance: 5 mm
- 15 kV
- Number of completed frames: 541
- Total counts: Range, but up to 340 million
- Process Time: 6
- Live time: up to 42600 seconds

**Parameters for FIB-SEM nanotomography runs**

**Soil tunnels FIB trench 2**

Using Zeiss Microscopy Atlas 5 (3D) software

- Pt pad: 15 x 15 µm
- 20 µm depth
- Registration marks: 700pA probe
- Deposition of first pad: Pt – 700pA probe
- Registration marks milling: REF beam and I beam depo (GIS)
- Upper Pt pad: 700pA probe
- Mill coarse trench: 7nA probe
- Polish cross section face: 700pA probe
- For run: 700pA probe

*Run setup:*

- Analytic mode, 1.8kV, 300pA, SESI only
- Brightness: 50.4
- Contrast: 28.2
- FOV: 28.17 µm
- Slice thickness: 10 nm
- Pixel size: 27.5 nm
- Dwell time: 7.0 µs
- Line averaging: 5 seconds
- Image size: 1024x1024 pixels
- Frame time: 42.95 seconds
- 27.51 x 27.51 nm spacing

**Voxel is 27.5 x 27.5 x 10 nm**

**Soil crusts FIB trench 1**

Using Zeiss Microscopy Atlas 5 (3D) software

- Pt pad: 7 x 10 µm
- 15 µm depth
- Registration marks: 700pA probe
- Deposition of first pad: Pt – 700pA probe
- Registration marks milling: REF beam and I beam depo (GIS)
- Upper Pt pad: 700pA probe
- Mill coarse trench: 7nA probe
- Polish cross section face: 700pA probe
- For run: 700pA probe

*Run setup:*

- Analytical mode, 1.8kV, 300Pa, SESI only
- FOV: 17.71 µm
- Brightness: 40.2
- Contrast: 50.1
- 17.29 x 17.29 nm spacing
- Dwell time: 12 seconds
- Line averaging: 12 seconds
- Slice thickness: 10nm

**Voxel is 17.3 x 17.3 x 10 nm**
